# Impact of Prior Biologic Exposure on Upadacitinib Efficacy in Inflammatory Bowel Disease: A Systematic Review of Randomized Controlled Trials

**DOI:** 10.7759/cureus.113539

**Published:** 2026-07-28

**Authors:** May Htun, Anupriya Lilhori, Mariam Alamgir, Marem Al-Shemary, Ritvik Sriram, Nejma Azeez, Pradeep Rajbhandari, Aiken Muratova, Siril Patil, Prabhav Kashyap Godavarthy, Ahmad Mahmood, Jannush Kylanathan, Advait Teli

**Affiliations:** 1 Medicine, University of Medicine Mandalay, Mandalay, MMR; 2 Internal Medicine, JSS Medical College and Hospital, New Delhi, IND; 3 Medicine, St. George’s University, True Blue, GRD; 4 Internal Medicine, Medical University of Lublin, Lublin, POL; 5 Medicine, Tirunelveli Medical College, Tirunelveli, IND; 6 Acute Medical Unit, East Sussex Health Care NHS Trust, Eastbourne District General Hospital, Eastbourne, GBR; 7 Public Health, University of New Haven, West Haven, USA; 8 Otolaryngology - Head and Neck Surgery, Kathmandu University School of Medical Sciences, Dhulikhel Hospital, Kathmandu, NPL; 9 Internal Medicine, AdventHealth Orlando, Orlando, USA; 10 Anesthesia and Critical Care, Fortis Hospital, Mulund, Mumbai, IND; 11 Obstetrics and Gynaecology, Mamata Academy of Medical Sciences, Hyderabad, IND; 12 General Surgery, Xinjiang Medical University, Ürümqi, CHN; 13 Medicine, University Hospital Limerick, Limerick, IRL; 14 Medicine, Bharati Vidyapeeth (Deemed to be University) Medical College, Pune, IND

**Keywords:** biologic therapies, clinical remission, crohn’s disease (cd), inflammatory bowel disease, janus kinase (jak) inhibitor, ulcerative colitis (uc), upadacitinib

## Abstract

Whether prior biologic exposure attenuates the efficacy of upadacitinib (UPA), a selective JAK-1 inhibitor, in inflammatory bowel disease (IBD) remains unresolved. This systematic review addresses this question across the induction and maintenance phases of ulcerative colitis (UC) and Crohn’s disease (CD). PubMed/MEDLINE, ScienceDirect, Scopus, and ClinicalTrials.gov were searched through from inception to January 2026 per Preferred Reporting Items for Systematic Reviews and Meta-Analyses (PRISMA) 2020 (PROSPERO: CRD420261287632). Eligible studies were Phase 3 randomised controlled trials (RCTs) in moderate-to-severe UC or CD reporting subgroup data by biologic exposure status. Risk of bias was assessed with Cochrane RoB 2; narrative synthesis followed the Synthesis Without Meta-analysis (SWiM) framework. Three studies with six Phase 3 programs were included, all at low overall risk of bias. While therapeutic response rates were numerically lower in patients with prior biologic exposure, UPA demonstrated sustained therapeutic benefit observed across all subgroups. In Crohn's disease, stratified risk differences (RD) for clinical remission consistently favored UPA over placebo (RD range: 14.5-33.0 pp). While biologic-naive patients achieved numerically higher remission rates, biologic-experienced patients retained meaningful clinical and endoscopic benefit at one year. In ulcerative colitis, UPA demonstrated superior efficacy across all primary endpoints compared to placebo. Safety data revealed a dose-dependent increase in adverse events (e.g., herpes zoster, hepatic enzyme elevations) at 30 mg compared to 15 mg in the maintenance phase. UPA provides clinically meaningful remission in biologic-experienced IBD patients across both UC and CD. Although prior biologic exposure is associated with lower absolute remission rates, the sustained therapeutic benefit supports UPA as a viable salvage option. Further research with head-to-head trials is needed.

## Introduction and background

Inflammatory bowel disease (IBD), which includes ulcerative colitis (UC) and Crohn’s disease (CD), is a chronic, immune-mediated condition of the gastrointestinal tract affecting millions of people worldwide, and incidence is on the rise in newly industrialized regions [1]. Anti-tumor necrosis factor (anti-TNF) drugs, integrin inhibitors, and IL-12/23 antagonists have been shown to be effective in reducing the incidence of moderate-to-severe disease [2]. Yet there are 10-30% of patients who do not respond to primary treatment, and of the patients who respond to initial therapy (up to 23-46%), a second loss of response occurs within one year [3-5]. Consequently, for the growing population of patients who have exhausted one or more lines of advanced therapy, evidence regarding optimal sequencing and positioning remains limited.

In the management of IBD, patients are broadly categorized as “biologic-naive” (having no prior exposure to targeted biologic therapies) or “biologic-experienced” (having prior exposure followed by biologic failure, intolerance, or secondary loss of response). Biologic-experienced patients often present with a more refractory disease phenotype or a treatment-resistant immunological profile, which can attenuate the efficacy of subsequent advanced therapies.

Upadacitinib (UPA) is an oral selective Janus kinase 1 (JAK-1) inhibitor. UPA has been shown to be superior to placebo in several Phase 3 randomised controlled trials (RCTs) in UC and CD to induce and maintain remission. Subgroup data from the previous studies also reveal that the effectiveness is maintained in biologic-experienced patients [5]. Although subgroup analyses indicate that UPA remains effective in patients with prior biologic exposure, the extent to which previous biologic use modifies therapeutic response in UC and CD has not been systematically quantified.

This is an important clinical distinction. In IBD, treatment algorithms are increasingly informed by prior therapy history, and to place a new agent in clinical practice, we need to know not only the efficacy of the treatment but also the safety of the treatment in patients with previously treated outcomes. There is currently no systematic review of UPA in biologic-naive and biologic-exposed patients in UC and CD and induction and maintenance. This systematic review fills this gap by providing a qualitative review of available Phase 3 RCT evidence to assess whether prior biologic exposure affects UPA's efficacy and its safety across the treatment spectrum.

## Review

Methods

Study Design and Registration

This systematic review was conducted in accordance with the Preferred Reporting Items for Systematic Reviews and Meta-Analyses (PRISMA) 2020 guidelines [6]. The study protocol was prospectively registered with PROSPERO prior to data extraction (Registration Number: CRD420261287632).

Search Strategy, Study Selection, and Eligibility Criteria

A systematic literature search was performed across PubMed/MEDLINE, ScienceDirect, Scopus, and ClinicalTrials.gov from inception through January 2026. No language restrictions were applied. The search strategy combined Medical Subject Headings (MeSH) terms and free-text keywords as follows: ("Upadacitinib" OR "Rinvoq" OR "ABT-494" OR "JAK-1 inhibitor") AND ("Ulcerative Colitis" OR "Crohn's Disease" OR "Inflammatory Bowel Disease").

The studies were eligible to be included if they met the following pre-specified eligibility criteria: published, original research from Phase 3 RCTs of UPA in adult patients aged 18 years or older with moderate-to-severe UC or CD; the addition of a placebo comparator arm; at least one pre-specified efficacy outcome (clinical remission or endoscopic remission) or safety outcome (serious adverse events or discontinuation rate) of the group; and the report of, or the potential to provide such subgroup efficacy information based on prior biologic exposure status (biologic-naive vs biologic-experienced) within the primary publication or the accompanying registry documentation. The studies were excluded if they were Phase 2 exploratory or dose-ranging trials, observational or registry-based in design, case reports or series, restricted to pediatric patients, and no placebo comparator arm was provided.

Data Extraction

Two independent reviewers screened titles, abstracts, and full texts against the eligibility framework, with a senior third reviewer resolving discrepancies via consensus. Data extraction captured trial program identity, design characteristics, intention-to-treat (ITT) denominators (including advanced therapy exposure history), primary efficacy endpoints, and adverse event frequencies with corresponding 95% confidence intervals (CIs). Quality assessment was executed using the Cochrane Risk of Bias 2 (RoB 2) tool for randomized trials [7]. 

Data Synthesis

Due to the clinical and methodological outcome definition variations between the disease states, the quantitative pooling was not done via meta-analysis. Specifically, substantial heterogeneity in trial designs, differing definitions of maintenance entry criteria, variable washout periods following prior biologic failure, and inconsistent time points for primary endpoint assessment precluded valid statistical integration. Therefore, the narrative synthesis was done adhering to the Synthesis Without Meta-analysis (SWiM) reporting framework [8]. 

To enhance clinical clarity, we extracted trial-level data to calculate unadjusted risk differences (RD) and 95% CI for efficacy outcomes in CD. These estimates were generated using R software (R Foundation for Statistical Computing, Vienna, Austria) to create descriptive forest plots, which visualize the therapeutic gain of UPA relative to placebo across trials without pooled meta-analytic summary. Findings for UC were assessed qualitatively and reported in a structured tabular format.

The main synthesis objective was to compare clinical remission rates between biologic-naive and biologic-exposed subgroups in induction and maintenance phases in UC and CD. Treatment gain, defined as the absolute difference in remission rate of UPA and placebo, was the standard of therapeutic benefit. Safety data were reported in a descriptive manner, considering dose-dependent differences in adverse event frequency and discontinuation rates between 15 mg and 30 mg maintenance groups.

Results

Study Selection and Characteristics

**Figure 1:**
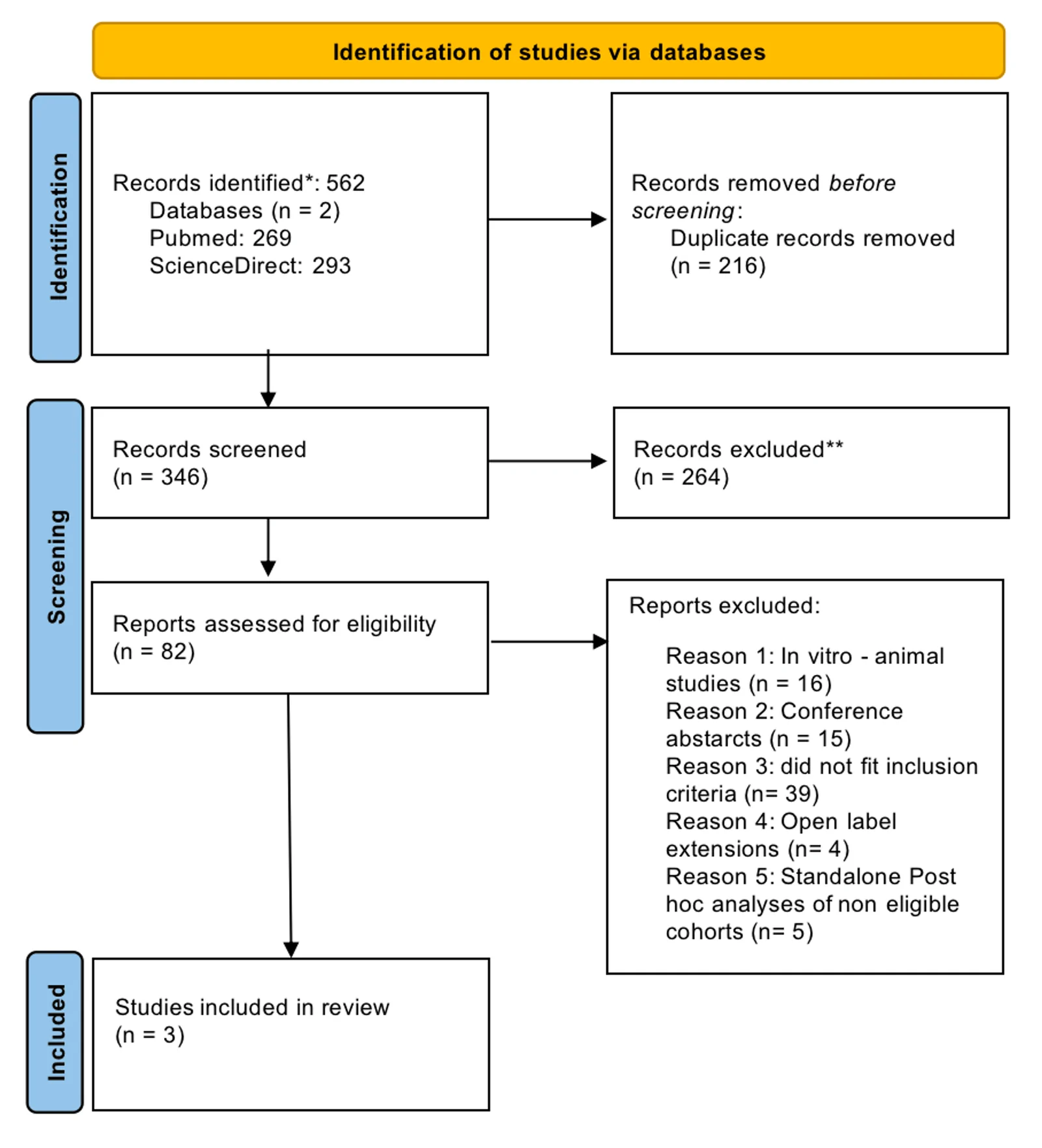
Preferred Reporting Items for Systematic Reviews and Meta-Analyses (PRISMA) Flow Diagram of the Study Selection Process. Flow diagram illustrating the systematic identification, screening, and selection of randomized controlled trials for inclusion in the systematic review. The search strategy identified 562 records, resulting in the inclusion of three randomized controlled trials comprising six Phase 3 clinical programs (U-ACHIEVE, U-ACCOMPLISH, U-ACHIEVE Maintenance, U-EXCEL, U-EXCEED, and U-ENDURE) [9-11].

The systematic search identified a total of 562 unique records. After removal of duplicates and screening of titles and abstracts, 82 full-text articles were assessed for eligibility. As detailed in the PRISMA flow diagram (Figure 1), three RCTs comprising six different Phase 3 clinical programs for UPA in IBD were included: U-ACHIEVE and U-ACCOMPLISH (UC induction), U-ACHIEVE Maintenance (UC maintenance), U-EXCEL and U-EXCEED (CD induction), and U-ENDURE (CD maintenance) [9-11]. Across all six programs, enrolled populations comprised adults with moderate-to-severe disease activity and varying degrees of prior biologic exposure, providing robust subgroup data for the primary synthesis objective. Detailed baseline characteristics for these studies are summarized in Table 1.

**Table 1:** Combined Baseline Characteristics (Ulcerative Colitis and Crohn's Disease Trials) *N represents the total number of participants in the intention-to-treat (ITT) population for each trial arm. In instances where the sum of demographic subgroups (sex) is less than the total N, the difference represents participants who were included in the primary ITT efficacy analysis but lacked documented baseline demographic data. **Age for maintenance studies is reported as median (interquartile range); all other age data are reported as mean (standard deviation). †Number of prior biologic exposure reported as >=2 ††Patients in U-EXCEED were required to be biologically experienced; therefore, prior biologic failure was 100%. UC: ulcerative colitis, CD: Crohn’s disease, UPA: upadacitinib

Trial	Disease	Arm	N*	Female, n (%)	Age, yr [Mean (SD)]	Prior Bio Failure, n (%)	Number of previous biologic failure (n)			
							1, n (%)	2, n (%)	≥3, n (%)	
U-ACHIEVE Induction [9]	UC	Placebo	155	57 (37%)	44.5 (23.0)	78 (51%)	29 (19%)	31 (20%)	22 (14%)	
		UPA 45mg	319	121 (38%)	43.0 (23.0)	168 (53%)	64 (20%)	64 (20%)	46 (14%)	
U-ACCOMPLISH Induction [9]	UC	Placebo	177	67 (39%)	42.0 (24.0)	89 (51%)	39 (22%)	36 (21%)	18 (10%)	
		UPA 45mg	345	127 (37%)	40.0 (24.0)	172 (50%)	64 (19%)	67 (20%)	42 (12%)	
U-ACHIEVE Maintenance [10]	UC	Placebo	223	100 (45%)	40 (32-54)**	116 (52%)	45 (39%)	71 (61%)†		
		UPA 15mg	225	79 (35%)	39.0 (30-53)**	109 (48%)	43 (39%)	66 (61%)†		
		UPA 30mg	233	92 (39%)	42.0 (31-55)**	111 (48%)	40 (36%)	71 (64%)†		
U-EXCEL Induction [11]	CD	Placebo	176	82 (46.6%)	39.3 (13.6)	78 (44.3%)	28 (35.9%)	24 (30.8%)	26 (33.3%)	
		UPA 45mg	350	161 (46.0%)	39.7 (13.7)	161 (46.0%)	58 (36.0%)	52 (32.3%)	51 (31.7%)	
U-EXCEED Induction [11]	CD	Placebo	171	75 (43.9%)	37.5 (12.1)	171 (100%)††	-	-	-	
		UPA 45mg	324	155 (47.8%)	38.4 (13.7)	324 (100%)††	-	-	-	
U-ENDURE Maintenance [11]	CD	Placebo	165	77 (46.7%)	38.1 (13.0)	126 (76.4%)	52 (41.3%)	32 (25.4%)	42 (33.3%)	
		UPA 15mg	169	67 (39.6%)	38.1 (13.5)	124 (73.4%)	52 (41.9%)	31 (25.0%)	41 (33.1%)	
		UPA 30mg	168	75 (44.6%)	37.0 (13.3)	127 (75.6%)	43 (33.9%)	35 (27.6%)	49 (38.6%)	

Upadacitinib Efficacy in Ulcerative Colitis

Table 2 presents overall efficacy outcomes in UC. During the induction phase of the study, UPA 45 mg had superior efficacy over placebo across all endpoints. Not only was clinical remission achieved by 21.6% (95% CI: 15.8-27.4) of patients in UC1 and 29.0% (95% CI: 23.2-34.7) in UC2, but objective measures such as endoscopic remission (12.7% in UC1; 15.9% in UC2) and histological-endoscopic mucosal improvement (23.7% in UC1; 30.1% in UC2) were also superior.

**Table 2:** Overall Efficacy of Upadacitinib Across Ulcerative Colitis Trials All values represent the adjusted difference (%) versus placebo with 95% confidence intervals (CI). Data for induction trials (UC1/UC2) reflect outcomes at week 8; data for the maintenance trial (UC3) reflect outcomes at week 52 in the intention-to-treat population. FACIT-F: Functional Assessment of Chronic Illness Therapy-Fatigue [12], LSM: least squares mean, PBO: placebo, QD: once daily, UC1: U-ACHIEVE Induction study, UC2: U-ACCOMPLISH Induction study, UC3: U-ACHIEVE Maintenance study, UPA: upadacitinib

Endpoint	UC1 (Induction) UPA 45 mg QD vs PBO [9]	UC2 (Induction) UPA 45 mg QD vs PBO [9]	UC3 (Maintenance) UPA 15 mg QD vs PBO [10]	UC3 (Maintenance) UPA 30 mg QD vs PBO [10]
Clinical remission (Adapted Mayo)	21.6% (15.8-27.4)	29.0% (23.2-34.7)	-	-
Endoscopic remission	12.7% (8.4-17.0)	15.9% (11.4-20.3)	18.6% (12.2-25.0)	21.9% (15.4-28.5)
Maintenance of clinical remission (Adapted Mayo)	-	-	34.9% (21.2-48.5)	46.9% (34.0-59.8)
Corticosteroid-free clinical remission	-	-	33.7% (20.0-47.3)	45.5% (32.6-58.5)
Maintenance of endoscopic improvement	-	-	42.2% (30.4-53.9)	51.5% (40.9-62.1)
Histological-endoscopic mucosal improvement	23.7% (17.5-30.0)	30.1% (24.1-36.2)	28.5% (21.1-35.9)	43.8% (36.1-51.5)
Mucosal healing	9.7% (5.7-13.7)	11.3% (7.2-15.3)	13.5% (7.8-19.3)	17.2% (11.2-23.3)
Change from baseline in FACIT-F score (LSM)	6.7% (4.8-8.6)	6.0% (4.2-7.7)	5.5% (3.5-7.5)	7.2% (5.3-9.2)

During the maintenance phase of the study, therapeutic benefits were seen across both 15 mg and 30 mg doses. Notably, a dose-dependent trend was observed with 30 mg achieving statistically significantly higher rates of remission across different parameters. Remission was preserved with profound histological-endoscopic mucosal improvement in the 30 mg cohort compared to the 15 mg cohort (43.8% [36.1-51.5] vs 28.5% [21.1-35.9]). Furthermore, patient-reported quality-of-life assessments mirrored these objective trends, with sustained improvements in fatigue as measured by the Functional Assessment of Chronic Illness Therapy-Fatigue (FACIT-F) score [12], with least squares mean (LSM) changes that favoured the higher 30 mg maintenance dose over the 15 mg regimen.

Upadacitinib Efficacy in Crohn's Disease

Table 3 presents efficacy outcomes in CD stratified by prior biologic exposure. Clinical outcomes were evaluated using a standard validated instrument, the Crohn's Disease Activity Index (CDAI) [13]. During the induction phase, UPA 45 mg demonstrated superior efficacy over placebo across both biologic-naive and biologic-experienced subgroups in both induction trials. In U-EXCEL, clinical remission was achieved by 54.3% of biologic-naive patients and 43.9% of biologic-experienced patients, with endoscopic response observed in 52.0% and 37.9%, respectively. In U-EXCEED, which enrolled an exclusively biologic-experienced population, clinical remission was achieved by 46.0% of patients with one or fewer prior biologic failures and 34.3% of patients with more than one prior failure, with endoscopic response rates of 47.6% and 26.3%.

**Table 3:** Overall Efficacy Outcomes for Upadacitinib in Crohn's Disease † No true treatment-naive subgroup exists for U-EXCEED (all patients had prior biologic failure by eligibility criteria); the rows here reflect "≤1" vs. ">1" prior biologics, not bio-naive vs. bio-exposed. CDAI: Crohn’s Disease Activity Index [13]; BIO-IR: biologic-immunomodulator refractory / biologic-experienced; Non-BIO-IR: biologic-naive; QD: once daily; pp: percentage points

Trial	Week	Subgroup	CDAI			Endoscopic response		
			Placebo %	Upadacitinib %	Risk Difference (pp)	Placebo %	Upadacitinib %	Risk Difference (pp)
U-EXCEL [11]	12	Non-BIO-IR (0 prior biologics)	39.8	54.3	14.5	16.3	52.0	+35.7
		BIO-IR (≥1 prior biologic)	15.6	43.9	28.3	9.0	37.9	+29.0
U-EXCEED† [11]	12	≤1 prior biologic failed	29.4	46.0	16.6	4.4	47.6	+43.2
		>1 prior biologic failed	15.5	34.3	18.8	2.9	26.3	+23.4
U-ENDURE [11]	52	Non-BIO-IR - 15 mg QD	25.5	46.7	21.2	17.9	39.8	+21.8
		Non-BIO-IR - 30 mg QD	25.5	56.1	30.6	17.9	43.9	+26.0
		BIO-IR - 15 mg QD	11.9	33.9	22.0	4.0	23.2	+19.2
		BIO-IR - 30 mg QD	11.9	44.9	33.0	4.0	38.9	+34.9

**Figure 2:**
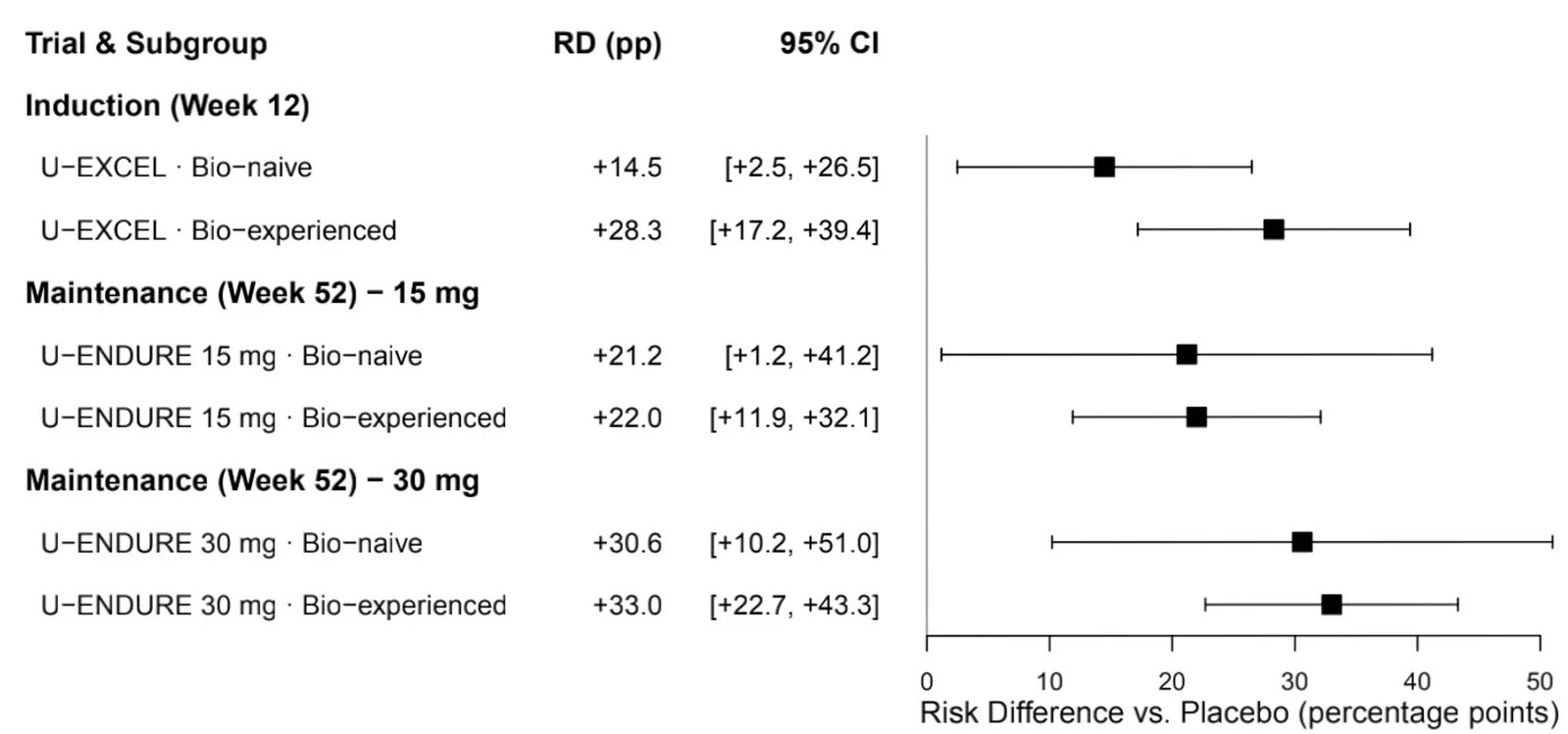
Forest Plot of Risk Differences for Clinical Remission (CDAI-Based) in Crohn’s Disease by Biologic Status. Risk difference (RD) in clinical remission (CDAI-based) between upadacitinib and placebo in biologic-naive and biologic-experienced subgroups. Data are presented as RD in percentage points (pp) with 95% confidence intervals (CI). Results are derived from the U-EXCEL induction trial (Week 12) and the U-ENDURE maintenance trial (Week 52) at 15 mg and 30 mg doses [11]. CDAI: Crohn’s Disease Activity Index [13]. Figure generated using R software (R Foundation for Statistical Computing, Vienna, Austria).

**Figure 3:**
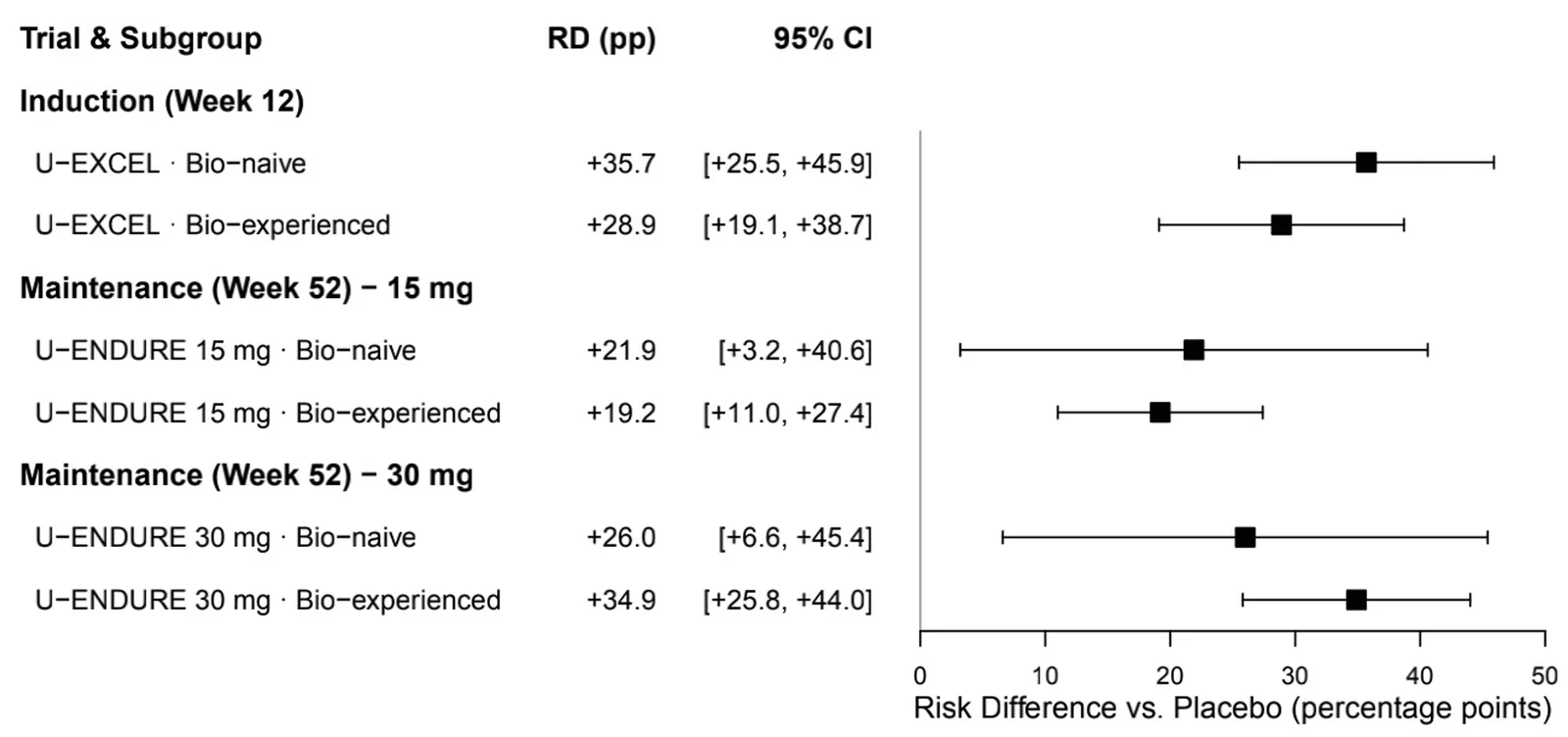
Forest Plot of Risk Differences for Endoscopic Response in Crohn’s Disease by Biologic Status. Risk difference (RD) in endoscopic response between upadacitinib and placebo in biologic-naive and biologic-experienced subgroups. Data are presented as RD in percentage points (pp) with 95% confidence intervals (CI). Results are derived from the U-EXCEL induction trial (Week 12) and the U-ENDURE maintenance trial (Week 52) at 15 mg and 30 mg doses [11]. Figure generated using R software (R Foundation for Statistical Computing, Vienna, Austria).

The therapeutic gain of UPA is further illustrated in Figure 2 and Figure 3, which visualize the RDs and 95% CIs across all subgroups. During the maintenance phase, therapeutic benefit was observed across both the 15 mg and 30 mg doses, with a clear dose-dependent trend. The 30 mg dose achieved higher rates of clinical remission than the 15 mg dose in both biologic-naive (56.1% vs. 46.7%) and biologic-experienced (44.9% vs. 33.9%) patients. This dose-dependency was mirrored in endoscopic response, where the 30 mg dose outperformed the 15 mg dose in both biologic-naive (43.9% vs. 39.8%) and biologic-experienced (38.9% vs. 23.2%) subgroups. As shown in Figure 2 and Figure 3, the RDs consistently favored UPA across all induction and maintenance cohorts, confirming that efficacy is maintained regardless of prior biologic exposure, albeit with varying degrees of therapeutic gain.

Safety and Tolerability

Table 4 summarizes adverse events and discontinuation rates across all included Phase 3 trials. The safety profile of UPA was entirely consistent with the class of selective JAK-1 inhibitors throughout all trial phases.

**Table 4:** Safety Profile and Discontinuation Rates of Upadacitinib Across Phase 3 Trials UPA: upadacitinib, UC: ulcerative colitis, CPK: creatine phosphokinase.

Trial	Dose	Common Adverse Events	Discontinuation Rate
U-ACHIEVE Induction [9]	UPA 45 mg	Nasopharyngitis, acne, CPK elevation, neutropenia (each ~ 5%)	2%
	Placebo	Worsening UC (14%)	9%
U-ACCOMPLISH Induction [9]	UPA 45 mg	Acne (7%)	2%
	Placebo	Worsening UC & headache (5%)	5%
U-ACHIEVE Maintenance [10]	UPA 15 mg	Hepatic disorder (17%)	5.5%
	UPA 30 mg	Hepatic disorder, CPK elevation (9.2-10.1%)	8.7%
	Placebo	Anemia (14.1%)	19.3%
U-EXCEED Induction [11]	UPA 45 mg	Nasopharyngitis, anemia (6.8-7.1%)	5.6%
U-EXCEL Induction [11]	UPA 45 mg	Anemia (8%)	4.3%
U-ENDURE Maintenance [11]	UPA 15 mg	Anemia (9.8%)	12.4%
	UPA 30 mg	Hepatic disorder (9.6%)	7.9%
	Placebo	Anemia (11.7%)	7.2%

During induction, UPA 45 mg was well tolerated across both UC and CD trials, with low discontinuation rates (2-5.6%) and an adverse event profile dominated by non-serious events including acne, nasopharyngitis, and transient creatine phosphokinase (CPK) elevations. Notably, discontinuation rates in UPA-treated patients were consistently lower than in placebo arms during induction, driven by higher rates of primary disease worsening in the placebo groups.

During maintenance, a distinct dose-dependent increase in adverse event frequency was observed at the 30 mg dose compared to 15 mg, most notably for herpes zoster (7.3% vs. 3.9% in U-ENDURE), hepatic enzyme elevations (9.2-9.6% across maintenance cohorts), and lymphopenia. Despite this, overall discontinuation rates remained lower in UPA maintenance arms than in placebo across most trials, reflecting the clinical burden of uncontrolled disease in the placebo group. Long-term extension data showed improved tolerability over time, with discontinuation rates dropping to 3-4%, suggesting that adverse events in the early maintenance period do not translate to sustained treatment burden.

In addition to common non-serious adverse events, clinically important safety events of special interest, including serious infections, herpes zoster, hepatic disorders, and hematologic abnormalities, were noted across all Phase 3 trials. Serious infections occurred at low rates but showed a dose-dependent increase during the maintenance phase, particularly in the 30 mg cohorts. Conversely, high-consequence class-specific events such as major adverse cardiovascular events (MACE), venous thromboembolism (VTE), and malignancies were infrequent, occurring at low and comparable rates between UPA and placebo arms across the induction and maintenance programs, consistent with the established safety profile of selective JAK-1 inhibitors.

Risk of Bias Assessment

Risk of bias was evaluated using the Cochrane RoB 2 tool across five core domains (Table 5) [7]. All five Phase 3 trials were judged to be at low overall risk of bias, confirming high methodological rigor. The U-ACHIEVE Maintenance trial raised some concerns in Domain 4 (outcome measurement), which is directly attributable to the open-label re-randomization design used during the maintenance phase transition, though this did not compromise the overall low risk judgment.

**Table 5:** Risk of Bias Assessment Using the Cochrane RoB 2 Tool [7]

Trial	D1 Randomization	D2 Deviations	D3 Missing Data	D4 Outcome Measurement	D5 Reporting	Overall Judgment
U-ACHIEVE (Induction) [9]	Low Risk	Low Risk	Low Risk	Low Risk	Low Risk	Low Risk
U-ACCOMPLISH [9]	Low Risk	Low Risk	Low Risk	Low Risk	Low Risk	Low Risk
U-ACHIEVE (Maint.) [10]	Low Risk	Low Risk	Low Risk	Some Concerns	Low Risk	Low Risk
U-EXCEL (Induction) [11]	Low Risk	Low Risk	Low Risk	Low Risk	Low Risk	Low Risk
U-EXCEED (Induction) [11]	Low Risk	Low Risk	Low Risk	Low Risk	Low Risk	Low Risk
U-ENDURE (Maint.) [11]	Low Risk	Low Risk	Low Risk	Low Risk	Low Risk	Low Risk

Discussion

The results of this systematic review show that prior biologic exposure can affect the clinical remission rate by 11-13%. However, there is still clinical benefit over placebo in UC and CD, as highlighted by the individual trial risk ratio ranging from 2.99 to 6.65 in this highly refractory population [9-11,14]. The attenuation of response in patients with prior biologic exposure is not an issue of UPA alone but a general trend that has been observed across the spectrum of IBD treatment in refractory populations. In vedolizumab's GEMINI 2 trial in CD, clinical remission at week six was 17.5% in anti-TNF-naive patients versus 10.5% in anti-TNF-experienced patients [15]. Similarly, for ustekinumab in UNIFI the induction clinical remission advantage over placebo was 18.4% in the biologic-naive UC patients and 12.7% in the biologic-experienced patients [16]. Sequential biologic strategies in anti-TNF-experienced CD patients yield only 24% of remission on a second biological agent versus placebo, and no single agent is better, as demonstrated by network meta-analysis [17]. Taken together, these benchmarks suggest UPAs biologic-experienced remission rates of 42-49% at one year in UC and CD, as noted in our review, are favourable clinical outcomes relative to the refractory population in which they were achieved.

Extending this observation across the treatment timeline, the difference in clinical response between biologic-naive versus biologic-experienced gap was not static but showed a temporal convergence as patients transitioned from induction to maintenance. In UC, the differential between biologic-naive and biologic-experienced patients narrowed from approximately 13% at Week 8 to less than 5% at Week 52 [9,10]. This pattern is consistent with the meta-analyses on UPA in the UC population, where refractory patients showed attenuated clinical and endoscopic response between Weeks 12 and 16 [18,19]. Therefore, these trial data highlight that therapeutic benefits may evolve over the course of the treatment following a delayed induction response, though further real-world evaluation is warranted.

The comparative positioning of UPA is further supported by evidence from network meta-analyses. A network meta-analysis of 35 RCTs found that UPA, tofacitinib, and ustekinumab were all highly ranked for remission in patients with prior biologic exposure, with approximately 70% of UPA-treated biologic-experienced patients achieving remission with induction therapy at a reference placebo rate [20]. As highlighted by Bressler et al., UPA can be effectively utilised early in the treatment algorithm for moderate-to-severely active UC in both biologic-naive and biologic-experienced populations, with comparative data suggesting favourable relative differences against certain alternative therapies, including vedolizumab and ozanimod [21]. In CD, a network meta-analysis of endoscopic outcomes found that JAK-1 inhibitors were the most effective for endoscopic remission overall, and that in biologic-experienced patients, JAK-1 inhibitors, anti-IL23p19 agents, and anti-IL12/23p40 agents were all superior to placebo for endoscopic remission [22].

Real-world evidence corroborates these trial findings across both disease contexts. In a recent multicentre study of anti-TNF-refractory Italian patients with UC (including patients who were non-respondent, intolerant, or in whom anti-TNF was contraindicated), JAK inhibitors including tofacitinib, filgotinib, and UPA were all effective; however, UPA achieved higher rates of clinical, steroid-free, and endoscopic remission at 52 weeks, with numerically higher adverse events associated with tofacitinib [23]. These findings support the use of UPA over earlier-generation JAK inhibitors in this population. In CD, a single-centre study in Chinese patients demonstrated clinical and biomarker remission with UPA at 12 and 24 weeks [24], while a French multicentre study showed that approximately one quarter of patients achieved clinical remission of perianal CD at one year [25]. Although these real-world data are limited by sample size, single-centre design, and retrospective methodology, their directional consistency with the Phase 3 RCT programme strengthens confidence in the external validity of the trial findings.

A clinically actionable observation emerging from both the systematic review and the real-world comparisons above is the potential role of dose optimisation. Endoscopic remission rates and quality-of-life improvements were numerically similar between the 15 mg and 30 mg maintenance doses in UC, despite higher clinical remission rates with 30 mg. This raises the prospect that dose de-escalation from 30 mg to 15 mg in patients who have achieved remission may be appropriate in order to reduce the dose-dependent adverse event profile, most notably herpes zoster and transient transaminase elevations, without meaningfully compromising endoscopic and quality-of-life outcomes [26]. Prospective studies specifically designed to evaluate structured de-escalation protocols are needed to formalise this approach.

Collectively, these findings suggest that positioning UPA earlier in the treatment sequence prior to multiple biologic failures may optimise therapeutic outcomes in both UC and CD. The drug's efficacy profile across induction and maintenance, its performance in biologic-experienced populations relative to class benchmarks, and its emerging real-world corroboration all support consideration of UPA as a first- or early second-line advanced therapy in appropriately selected patients. However, robust head-to-head trials against active comparators, rather than placebo-controlled data alone, are ultimately required to solidify treatment algorithms and guide positioning decisions with precision.

Limitations

There are several limitations to this review. First of all, no quantitative meta-analysis was conducted since the clinical and methodological differences between registration programs have been considered in the clinical remission indices used for UC (adapted Mayo score) and CD (CDAI score). SWiM is able to provide a qualitative synthesis of the study data but no pooled effect sizes have been identified as having a weighted effect size for the biologic-attenuation gap. Second, the data are derived in very controlled Phase 3 RCT settings where eligibility criteria are strict, so these results may not be representative of highly refractory, multi-morbid patients in clinical practice. Third, because the subgroup analyses by prior biologic exposure status were secondary endpoints in the trials, subtle differences within these populations could not be identified. Furthermore, the present data aggregate all biologic-experienced patients into a single group, preventing a granular evaluation of outcomes based on the specific number or mechanistic class (e.g., anti-TNF vs. anti-integrin) of prior failed advanced therapies. Finally, broader prospective registry data remain necessary to monitor long-term safety.

## Conclusions

This systematic review provides a structured synthesis of UPA efficacy stratified by biologic exposure status across both UC and CD. Prior biologic exposure attenuates but does not negate UPA's therapeutic benefit, and remission rates in biologic-experienced patients across induction and one-year maintenance are clinically meaningful and contextually favourable relative to published benchmarks for alternative advanced therapies. Furthermore, observed trial trends including outcome convergence over time and dose-dependent safety signals offer hypothesis-generating evidence to inform maintenance dosing strategies. However, as these findings are derived from indirect, cross-trial comparisons in the absence of head-to-head data, definitive treatment positioning requires validation through dedicated comparative trials and prospective real-world evaluations.
